# Modular intercalary prosthetic reconstruction for malignant and metastatic tumours of the proximal femur

**DOI:** 10.1038/s41598-024-56645-7

**Published:** 2024-03-11

**Authors:** Lu Liu, Xiao-qiang Deng, Yong-jie Zhao, Rong-xing Ma, Li Yang, Kun-xiu Song, Jing-yu Zhang, Yong-cheng Hu

**Affiliations:** 1 Graduate School, Tianjin Medical University, 22 Qixiangtai Road, Tianjin, China; 2 Xing’anmeng People’s Hospital, 66 Hanshan West Street, Ulanhot, Inner Mongolia China; 3 Binzhou Medical University Hospital, 661 Huanghe 2 Road, Binzhou, China; 4 Beijing Jishuitan Hospital Guizhou Hospital, 206 Sixian Street, Guiyang, Yunnan China; 5 Department of Bone Tumor and Soft Tissue Oncology, Tianjin Hospital, 406 Jiefang Southern Road, Tianjin, China

**Keywords:** Femur, Malignant, Prosthesis, Reconstructive surgery, Tumour, Oncology, Outcomes research, Bone cancer

## Abstract

To illustrate the surgical technique and explore clinical outcomes of the reconstruction for the malignant and metastatic bone tumour of proximal femur with metallic modular intercalary prosthesis. Sixteen patients who underwent modular intercalary prosthetic reconstruction after tumour resection were included from April 2012 and October 2020. Prosthesis and screws parameters, resected bone length and residual bone length, clinical outcomes and survivorship were analyzed. All patients were followed up for an average of 19 months (range 1–74). In our series, 12 patients died of the progression of the primary disease at the final follow-up. The cumulative survivorship since the treatment of proximal femoral metastasis was 78.6% (11 patients) at 6 months and 38.5% (5 patients) at 1 year. The mean MSTS score was 22.25 ± 4.55 among all patients. There were no cases of loosening or breakage of the prostheses, plates or screws, despite the various measurements of prostheses and residual bones. Modular intercalary prosthetic reconstruction was an effective method for malignant tumour of the proximal femur, including the advantages of providing early pain relief, quickly restoring postoperative function, required a short operation time, and preserving the adjacent joints.

## Introduction

Malignant bone tumours most often invade the proximal femur^1,2^. Most primary malignant bone tumours or metastases in the proximal femur require surgical treatment, especially those with pathological fractures. Limb salvage surgery has been improved along with great advances of neoadjuvant therapy. Limb salvage consist of two parts: tumour resection and structural bone defect reconstruction. Following tumour resection, many reconstruction methods are available for the treatment of malignant tumours of the proximal femur, such as prosthetic reconstruction^3^, massive allografts^4^, autogenous bone grafts^5^, and autogenous extracorporeally inactivated tumour-bearing bone^6^. However, the optimized surgical method for a variety of candidates with malignant tumour of the proximal femur is still a controversial, and reconstructive options in limb salvage, such as those involving massive allografts, autogenous bone grafts, and autogenous extracorporeally inactivated tumour-bearing bone, are not suitable for proximal femur reconstruction due to its particularity of anatomical structure and biomechanics^7,8^; the proximal femur has irregularly anatomical shape of trochanteric area, and transmits complex forces in various directions from pelvic to lower extremity. Prosthesis reconstruction, including modular total hip prosthetic replacement and hemiarthroplasty, are commonly used for the treatment of proximal femoral malignant tumours, as these methods with femoral head replacement favorably leads to the early recovery of limb function and requires a short operation time. However, total hip replacement and hemiarthroplasty compromises the hip joint, has a large resection range and requires extensive soft tissue dissection. Given excising the tumor involving the proximal femur, adjacent hip joint can be preserved, the surgery will lead to less trauma in patients, and enables an early return to daily activities^9–11^.

Intercalary prosthetic reconstruction has been increasingly used in recent years to treat the tumour located at the middle shaft of long bone, a length of proximal and distal prosthetic stem is necessary to guarantee enough stability of prosthesis with bone cement fixation^3^. However, this method has some disadvantages. Hanna et al. reported the outcomes of 23 patients treated with prosthetic reconstruction after the segmental resection of primary bone tumours, and the complications included breakage of the prosthesis (8%), aseptic loosening (4%), and periprosthetic fractures (4%)^12^. Sewell et al. reported the outcomes of 18 patients treated with intercalary diaphyseal prosthetic reconstruction for malignant tibial bone tumours, and the complications included four cases of aseptic loosening, and two cases of periprosthetic fracture^13^. Damron et al. reported the outcomes of 32 patients treated with intercalary diaphyseal prosthetic reconstruction for humeral bone tumours, and the complications included five cases of aseptic loosening^14^. Endoprosthesis is increasingly used for reconstruction after resection of proximal femur tumors, and is a good option. There have been a variety of reports on modular intercalary prosthetic reconstruction for the diaphysis of upper and lower extremities^15^, however, there is no one report specializing in this method for the proximal femur; there is insufficient data, and we must determine how to achieve better results.

The proximal femur is second common location of primary bone tumour, only less than about knee joint and significantly higher than proximal humerus. Given the intercalary prosthesis can be utilized more closer to the subtrochanteric area, the candidates can increase in a geometric ratio. Based on the 3-dimension finite element research, the tolerable shortest length of proximal stem of intercalary prosthesis in biomechanics for primary stabilization is not less than 5 cm in bone cement fixation. Otherwise, auxiliary components are added in design to ensure the proximal stability of prosthesis. Complete tumour removal is the basic requirement of limb salvage to achieve local control. However, the resection of tumor bearing bone is determined by several factors, such as malignancy, primary or secondary tumour, originating from bone or soft tissues, which result in some uncertainty in surgical excising process, especially the resection length and following structural defect of bone, modularity of intercalary prosthesis facilitate the surgical resection and increase of candidates. We designed an auxiliary plate and screws to improve the fixation stability of modular intercalary prosthesis with connecting mechanism of combine Morse tap and lap tap of components.

Therefore, we retrospectively analyzed the outcomes of modular intercalary prosthetic reconstruction for malignant bone tumours of the proximal femur. The aims of our study were (1) to illustrate our experience with the reconstruction and fixation method; (2) to evaluate the postoperative oncological results and functional results associated with modular intercalary prostheses for reconstruction after the removal of proximal femoral lesions; and (3) to evaluate the prosthesis-related complications.

## Results

### General information

All patients were followed up for an average of 19 months (range 1–74). The mean survival time was 19 months (range 1–74) for the 16 patients and 18.3 months (range 3–69) for the deceased at the final follow-up. One patient with a primary tumour and 3 patients with metastatic tumours were alive and did not have evidence of disease. Twelve patients died of the progression of the primary tumour, and there were no complications related to the prostheses in the 11 patients within the survival period. One patient (patient 7) with sciatic nerve injury of the left lower extremity preoperatively due to surgical complication of prior subtrochanteric traumatic fracture 20 years ago was followed up for only 1 month. The patient could not lift the limb or move the ankle for many years and could hardly walk with the aid of crutches. The patient recovered function postoperatively, and she could walk with a cane.

### EBL and duration of the procedure

The median bleeding volume was 887.5 ml. The least bleeding was 400 ml and the most bleeding was 2000 ml. The average operation time was (102.5 ± 26.8) min.

### Measurement results of prostheses and screws

**Figure 1:**
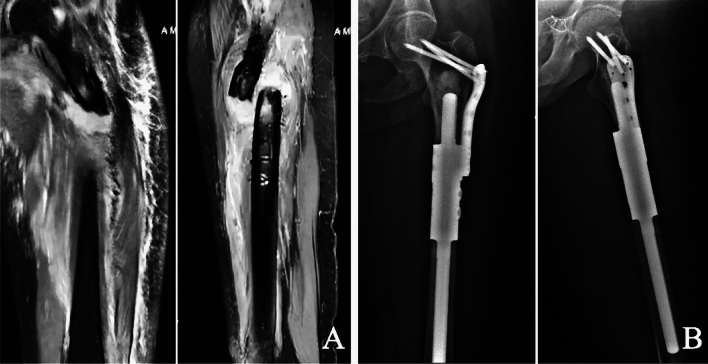
Cemented fixation of proximal femoral prosthesis in the 51-year-old woman above following resection of a metastatic carcinoma. (**A**) Fat-suppressed T2-weighted (T2W) images showing a pathological diaphyseal fracture which had intramedullary hematoma with fat necrosis and peripheral hematoma. (**B**) Anteroposterior and lateral radiographs showing that screws of the auxiliary lock plate were fixed in the femoral neck, and all screws were in the appropriate position.

**Figure 2:**
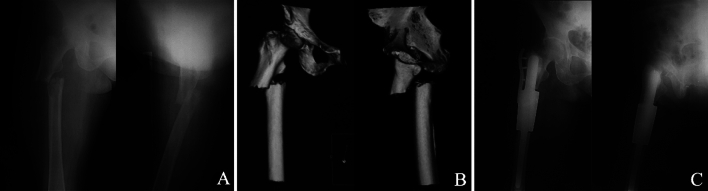
Cemented fixation of a proximal femoral prosthesis in a 53-year-old woman following resection of a metastatic breast carcinoma. (**A**) Anteroposterior and lateral radiographs showing a pathological diaphyseal fracture due to metastatic tumor. (**B**) 3D reconstruction by CT showing a pathological diaphyseal fracture, and fracture end was irregular. (**C**) Anteroposterior and lateral radiographs showing that screws of the auxiliary lock plate were fixed in the unilateral cortical.

**Figure 3:**
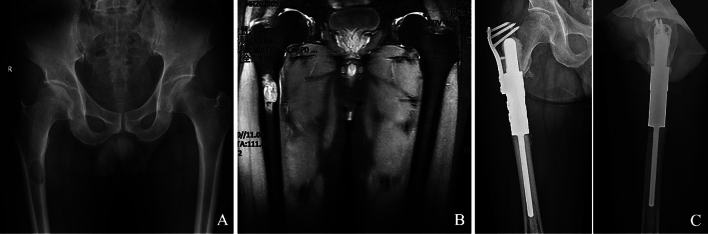
Cemented fixation of a proximal femoral prosthesis in a 58-year-old man following resection of a metastatic lung carcinoma. (**A**) Anteroposterior radiograph of the pelvis showing that there was an irregular low-density shadow below the greater trochanter of the right femur, and with obvious destruction of the bone cortex. (**B**) Fat-suppressed T2-weighted (T2W) image showing an irregular high-density shadow below the greater trochanter that with obvious destruction of the bone cortex of the right femur. (**C**) Anteroposterior and lateral radiographs showing that screws of the auxiliary lock plate were fixed through the greater trochanter.

The average length of the proximal stem was 6.36 ± 1.43 cm (range 2.8–9 cm), and the diameter was 1.54 ± 0.27 cm (range 1–2 cm). Proximal of auxiliary lock plate was fixed with 3 or 4 screws, and fixation through femoral neck (Type I) in 5 patients (31.25%) (Fig. 1), unilateral cortical fixation (Type II) in 7 patients (43.75%) (Fig. 2), fixation through greater trochanter (Type III) in 4 patients (25%) (Fig. 3). There were no complications about prosthesis with different prosthesis parameters and fixing form of the auxiliary lock plate.

### Measurement results of resected bone and residual bone

The mean length of resected bone was 11.30 ± 3.40 cm (range 6.5–18.2 cm). The mean distance from proximal end of the defect to lower edge of the lesser trochanter was 2.21 ± 1.62 cm (range 0–6 cm). The mean residual bone length of the proximal femur was 10.10 ± 2.35 cm (range 6.4–15 cm). The mean ratio of the proximal length of the stem to residual bone was 0.64 (range 0.44–0.8). There were no cases of loosening or breakage of prostheses, plates or screws, despite the various results above.

### Oncological outcomes

New metastases occurred in 6 patients postoperatively, including 3 of whom had lung metastases, 1 had liver metastasis and 2 had spinal metastases. All patients were managed by chemotherapy according to hospital databases and outpatient clinical records.

One patient with parosteal osteosarcoma experienced local recurrence 1 year after the operation, managed by amputation and died of the disease at 69 months postoperatively.

### Survivorship

**Figure 4:**
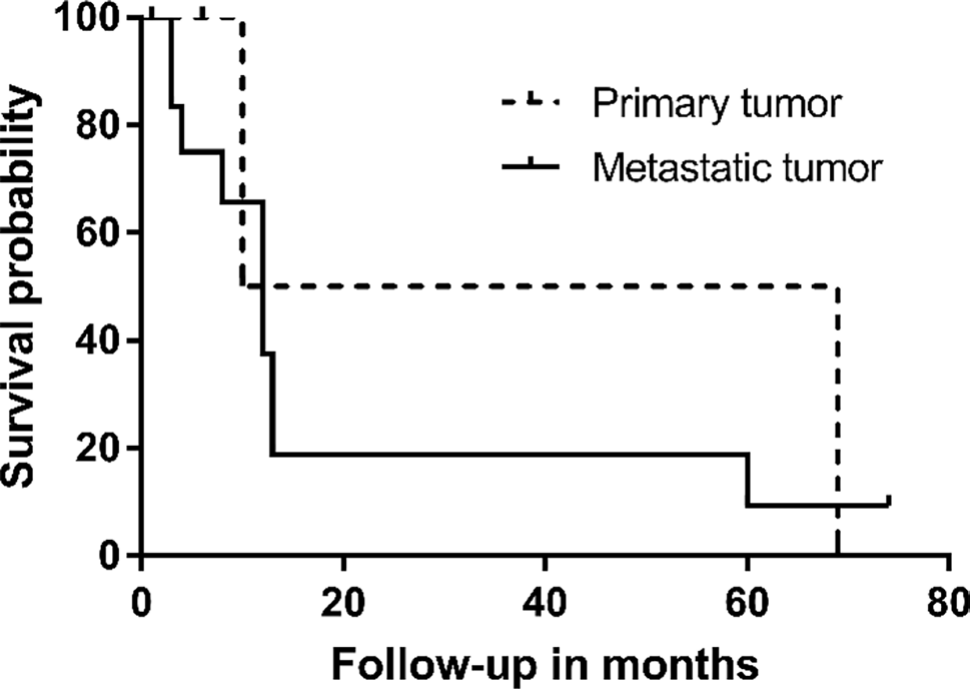
A Kaplan–Meier survival curve shows the survival of the patients with primary tumour was 100% at 6 months, 50% at 1 year; the survival of the patients with metastatic tumour was 72.7% at 6 months, 40% at 1 year.

Except for 2 patients who underwent surgery within 6 months, the cumulative survivorship of patient since the treatment of proximal femoral metastasis was 78.6% (11 patients) at 6 months and 38.5% (5 patients) at 1 year. The survivorship of patient with primary tumour was 100% (3 patients) at 6 months and 50% (1 patient) at 1 year, and for metastasis was 72.7% (8 patients) at 6 months and 40% (4 patients) at 1 year. The Kaplan–Meier actuarial curves was used to evaluate the survival (Fig. 4).

### Functional outcomes

#### MSTS score

At a mean of 2.5 months, 11 patients (68.8%) were able to walk without crutches, 4 patients (25%) needed to use a stick when walking, and 1 patient (6.2%) could not walk because of poor health. The mean MSTS score was 22.25 ± 4.55 among all patients. We concluded that modular intercalary prosthetic reconstruction can favorably improve the early function of patients.

#### VAS score

All patients exhibited improved quality of life resulting from a reduction in the severity of or the resolution of pain after surgery. The mean VAS score of all patients in our study was 8.6 ± 1.4 preoperatively, which improved to 3.1 ± 1.0 at day 3 after surgery and 1.3 ± 0.9 at 1 month. The statistical results showed a significant improvement in pain from preoperatively to 1 month (P < 0.05) postoperatively but not 3 days (P > 0.05).

### Complications

There was a very low complication rate of 6.25% with modular intercalary prosthetic reconstruction in our study, and only one patient had an amputation because of tumour progression (type V) one year after the operation according to Henderson et al.^16^ There were no other complications during the follow-up period, including soft tissue failure (type I), aseptic loosening (type II), structural failure (type III) and infection (type IV). In our study, fixing form of the auxiliary lock plate, resected bone length and residual bone length to the stability of prosthesis need to be further observed.

## Discussion

In our study, all 16 patients who underwent modular intercalary prosthetic reconstruction had good postoperative outcomes. This surgical method successfully relieved pain, preserved joint function, gained local control of the lesions, and led to few complications, thereby increasing the quality of life of patients^17^. Moreover, the treatment provided long-lasting relief of symptoms. According to the results of our study, no cases of loosening or breakage of the prostheses, plates or screws occurred, which may be related to the short follow-up time after implantation.

### Our experience with the reconstruction and fixation method

The proximal residual bone was relatively short after resection of the tumour when modular intercalary prosthetic reconstruction was performed for the proximal femur tumours. Therefore, the proximal stem of the prosthesis was short, especially when the osteotomy plane was at the lower edge of the lesser trochanter, and the medullary cavity above the lesser trochanter widens gradually, which may increase the probability of complications such as prosthesis loosening. Abudu et al. suggested that the shortest length suitable for fixation with prosthesis was 5 cm, but there was a risk of early loosening for fixation in such a short segment^18^. Sewell et al. suggested that an extracortical plate could be used to further strengthen the fixation when intramedullary fixation of the short-segment was < 4 cm^13^. We used an extracortical plate for all the patients in our study. Distal end of the plate was fixed on the prosthesis; the proximal end was fixed to the greater trochanter, unilateral cortical bone or femoral neck; and the stability of the prosthesis was high. Furthermore, we tried to fix the screws of the proximal plate in the femoral neck when the length of the proximal stem was > 4 cm and did not exceed the upper edge of the femoral neck. Screws fixed within the femoral neck can achieve better stability than those fixed outside of the femoral neck. There were no complications related to the prosthesis, regardless of the kind of fixation used in our study, which may be related to the short survival time of the patients.

### Evaluation of functional outcomes of the reconstruction

Because of the relatively short survival time of patients with malignant bone tumours, the surgery goals are to obtain early stability, enable early functional exercise and enable weight bearing after reconstruction to reduce the number of complications caused by bed rest, such as lower extremity venous thrombosis. Bus et al. reported a mean load-bearing time of 9 months in 44 patients who underwent massive allograft reconstruction^19^. Han et al. reported that the average time of weight bearing was 19 months in patients with autogenous fibula transplantation^20^. Benevenia et al. reported in a study that the average weight-bearing time of 21 patients undergoing femoral prosthesis reconstruction was 3 months^21^. In our study, the mean weight-bearing time of 15 patients (one patient was bedridden until death) was less than 2 months, and good function was observed for all the patients before began weight bearing tasks. The patients who died early could move with stability and without pain in the proximal femur during the last few months. The mean MSTS score in our study was 22.25 ± 4.55 points, which is comparable to those in previously published studies^12,22–25^. Only one patient had a lower score of 12 points because of a poor physical condition preoperatively, and the patient died within the third month postoperatively. All 4 patients who were alive at the last follow-up in our study were able to return to their preoperative lifestyle. No variables significantly influenced the functional outcomes, such as the resection length, type of tumour and presence/absence of a pathological fracture.

Patients with malignant bone tumours usually have severe pain that gradually worsens with the progression of the tumour, especially with a pathological fracture. The mean VAS score in our study was 8.6 ± 1.4 preoperatively, which improved to 3.1 ± 1.0 at day 3 after surgery and 1.3 ± 0.9 at 1 month. The pain of patients had been significantly improved at 1 month (P < 0.05) but not at 3 days (P > 0.05) compared with preoperatively. In our study, all of the patients exhibited early pain relief and were even completely pain free. In the early postoperative period, the patients could perform functional exercises with early pain relief even while walking, which considerably improved their quality of life.

### Evaluation of complications about the reconstruction

Modular intercalary prostheses have a long service life and can prevent problems such as poor long-term bone healing and host-donor nonunion. The main complications of modular intercalary prostheses are aseptic loosening and infection^21^. Ruggieri et al. reported in a study that the aseptic loosening rate was 17% after intercalary endoprosthesis reconstruction, and the average time of occurrence was 18.5 months^26^. Benevenia et al. reported that the aseptic loosening and infection rate was 14.6% in their study of 41 patients who received a total of 44 intercalary implants, and the mean time of occurrence was 14 months^21^. In our study, the rate of complications was lower (6.25%) than that in some previous studies, and only 1 patient underwent amputation because of disease progression one year after the operation. Eleven of the 12 patients who died had no complications, such as aseptic loosening, infection or breakage before death, and the 4 living patients did not have complications. The low complication rate in our study might be related to the short life expectancy that the patients have died prior to implant failure.

### Limitations

There are several limitations of our study. First, the follow-up duration after implantation was shorter due to the short life expectancy of most patients, and we could not evaluate the long-term effect accurately. Second, the sample size in our study was small. Third, biomechanical studies of the implant and proximal femur after reconstruction are lacking. Lastly, the difference between tumor type and primary tumor.

## Conclusions

Modular intercalary prosthetic reconstruction has the advantages of providing early pain relief, quickly restoring postoperative function, required a short operation time, simplifying the surgery process, and preserving the adjacent joints. It is a viable option for malignant tumours of the proximal femur.

## Methods

### Inclusion and exclusion criteria

Inclusive criteria: (1) patients with malignant tumour or pathological fracture of the proximal femur with subtrochanteric area involvement; (2) surgical therapy with tumour resection and reconstruction with modular intercalary prosthesis; (3) prosthetic measurement and clinical outcomes were evaluated; (4) a retrospective observational study, and (5) Life expectancy exceeds 6 months.

Exclusive criteria: (1) acute infection in the body; (2) patient with poor health to tolerate the surgery; (3) incomplete medical data, and (4) normal hip joint function without sever osteoarthritis or femoral head necrosis.

### Patient characteristics

Data were obtained from the hospital databases and outpatient clinical records. This was a retrospective review of 16 patients who underwent modular intercalary prosthetic reconstruction of the proximal femoral diaphysis between April 2012 and October 2020. There were 8 males (50%) and 8 females (50%), with a mean age of 63 years (range 32–82) at the time of operation. The diagnosis of patients was primary tumour of bone in 3 cases (18.8%, 3/16), including of parosteal osteosarcoma 1 case, spindle cell sarcoma 1 case and malignant bone lymphoma 1 case; and metastatic tumour of bone in 13 cases (81.2%, 13/16), including of breast carcinoma in 4 cases (30.8%, 4/13), lung carcinoma in 2 cases (15.4%, 2/13), renal carcinoma in 1 case, liver carcinoma in 1 case, and unknown origin tumour in 5 cases (38.5%, 5/13) (Table 1).

**Table 1:** The characteristics of included patients.

Case	Gender	Age (years)	Diagnosis	BMI	ECOG score	comorbidities	adjuvant treatment	Cement	Plate	Follow-up (months)	Patient status
1	M	82	MT	20.2	1	Hypertension	None	Y	Y	13	Dead
2	M	58	LC	23.2	1	None	None	Y	Y	4	Alive
3	F	52	BC	20.3	2	None	None	Y	Y	13	Dead
4	F	61	SCS	22.8	2	Cardiopathy	None	Y	Y	6	Alive
5	M	67	LC	22.5	2	None	None	Y	Y	3	Dead
6	F	66	BC	24.8	1	None	None	Y	Y	12	Dead
7	F	51	MT	22.9	1	None	None	Y	Y	1	Alive
8	F	57	LCA	17.6	1	None	None	Y	Y	4	Dead
9	F	32	POS	21.3	2	None	Chemotherapy	Y	Y	69	Dead
10	M	80	MT	21.3	2	Hypertension	None	Y	Y	12	Dead
11	M	63	RC	20.4	2	None	None	Y	Y	12	Dead
12	F	63	BC	22.7	2	None	None	Y	Y	60	Dead
13	F	57	BC	22.3	1	None	None	Y	Y	74	Alive
14	M	73	MT	22.6	2	None	None	Y	Y	3	Dead
15	M	76	MBL	23.2	2	None	None	Y	Y	10	Dead
16	M	71	MT	22.7	2	None	None	Y	Y	8	Dead

*MT* metastatic tumor, *LC* lung carcinoma, *BC* breast carcinoma, *SCS* spindle cell sarcoma, *LCA* liver carcinoma, *POS* parosteal osteosarcoma, *RC* renal carcinoma, *MBL* malignant bone lymphoma.

Thirteen patients (81.2%, 13/16) suffered from pathological fractures, including of primary tumour in 2 patients (15.4%, 2/13) and metastatic tumour in 11 patients (84.6%, 11/13). All patients received postoperative adjuvant chemotherapy and one patient received preoperative chemotherapy, which were obtained from the hospital databases, outpatient clinical records and telephone calls records. This retrospective study was performed in accordance with the ethical standards of the institutional and national research committee and with the 1964 Helsinki Declaration and its later amendments or comparable ethical standards. The study has been approved by the Human Investigation Committee of Tianjin hospital. All methods were implemented in accordance with the institutional guidelines and regulations. Informed consent was obtained from all individual participants included in the study.

### Imaging features

**Figure 5:**
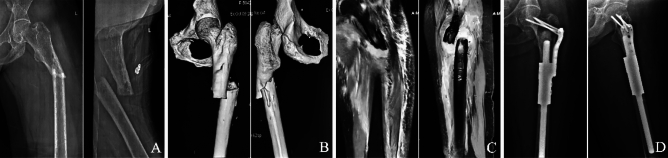
A female aged 51 years, left proximal femur metastasis from unknown origin. (**A**) Anteroposterior and lateral radiographs of a left femur showing a pathological diaphyseal fracture due to metastatic tumor. (**B**) 3D reconstruction by CT showing a pathological diaphyseal fracture, and fracture end was irregular. (**C**) Fat-suppressed T2-weighted (T2W) images showing a pathological diaphyseal fracture which had intramedullary hematoma with fat necrosis and peripheral hematoma. (**D**) Anteroposterior and lateral radiographs showing that modular intercalary prosthetic reconstruction after tumour resection.

Plain radiography (PR) revealed that all tumours involved the proximal femur and below the greater trochanter. Lytic neoplasm was observed in 15 patients according to the PR, and with obvious cortical destruction; osteoplastic tumor was observed in no patient. Pathological complete fracture was observed in 13 patients, and with obvious displacement including fracture ends were angled in 6 patients and overlapped in 7 patients (Fig. 5A). Computed tomography (CT) showed osteolytic destruction in 15 patients with low-density images, osteoblasts in 0 patients, and mixed osteoblasts in 1 patient with high-density images. All 15 patients of osteolytic destruction had different degrees of cortical destruction, and lesion margin was irregular. There was a soft tissue mass around the lesion in 9 patients (Fig. 5B). Magnetic resonance imaging (MRI) showed that an extra-osseous soft tissue component was observed in 9 patients (56.3%), tumour was confined to the femoral shaft in 7 patients (43.7%). Thirteen pathological fractures showed focal bone abnormalities, 10 of these (76.9%) had intramedullary hematoma with fat necrosis and peripheral hematoma, the other three cases (23.1%) had mixed signals around the fracture (Fig. 5C). PR revealed that all patients were reconstructed with modular intercalary prosthesis after tumor resection (Fig. 5D).

All surgical operations were performed by the senior doctor.

### Surgical procedure

#### Tumour resection

After general anesthesia, the patient was placed in lateral position. A longitudinal incision was made on the lateral thigh. Tumour resection was carried out according to the principles defined by Enneking et al.^27^ to achieve a safe excision 3 cm away from the response zone if possible in primary bone tumour. Specimens taken from the proximal and distal imprints were sent for histological examinations to confirm the type of tumour.

#### Medullary cavity preparation

The proximal and distal intramedullary canals were reamed to the appropriate size with rigid intramedullary reamers, beginning with a small size and ending with a size at least 2-mm larger than the implant to be implanted. This aimed to achieve a mantle of poly-methyl methacrylate around the stem with 1-mm.

#### Prostheses positioning

The prosthesis has reconstruction segment, an extension segment was used when the reconstruction segment was not long enough. The length of lesion was measured preoperatively and intraoperatively to decide whether to use the extension segment. Both stems were cemented in situ in the proximal and distal canals. Half of the lap tap was then placed onto each stem, the prostheses were then assembled and connected with two locking bolts placed through it. To avoid poor rotation and alignment of the diaphysis after reconstruction, we used an electrotome to mark the diaphysis for the proper lower limb alignment before the tumour was removed.

#### Auxiliary lock plate positioning

The auxiliary lock plate was fixed on the lateral side of the proximal reconstruction segment with two screws of 6 mm in diameter, and greater trochanter of femur needs to be covered. The proximal stem should be placed as close as possible to the proximal medullary cavity. According to the position of proximal stem at the apex of proximal femur, the fixation type of auxiliary lock plate was determined including fixation through femoral neck, unilateral cortical fixation and fixation through greater trochanter. Locking screws were used for unilateral cortical fixation, and cancellous screws were used for the others. An appropriate auxiliary lock plate was chosen to fix the proximal femur and enhance the stability of the prosthesis. It was important that set screws should be inserted quickly before the cement has fully hardened. The wound was then closed in layers after a suction drain was inserted.

### Postoperative management

All patients were treated with second-generation cephalosporins until the drainage tube was removed. Patients were allowed to move the affected limb at will immediately after the operation, and could leave the bed after pulling out the drainage tube. The patients were allowed to perform full weight bearing and range-of-motion exercises as tolerated with or without a brace after the operation.

### Follow-up

Routine follow-up was performed monthly for the first 3 months clinically and radiologically, followed by 3 months interval for the rest of the first year postoperatively, and the subsequent follow-up every 6 months. Clinical and radiological evaluations were performed at each follow-up to determine if there was local recurrence or distant metastasis, function or prosthesis problems.

### Outcomes measurement

**Figure 6:**
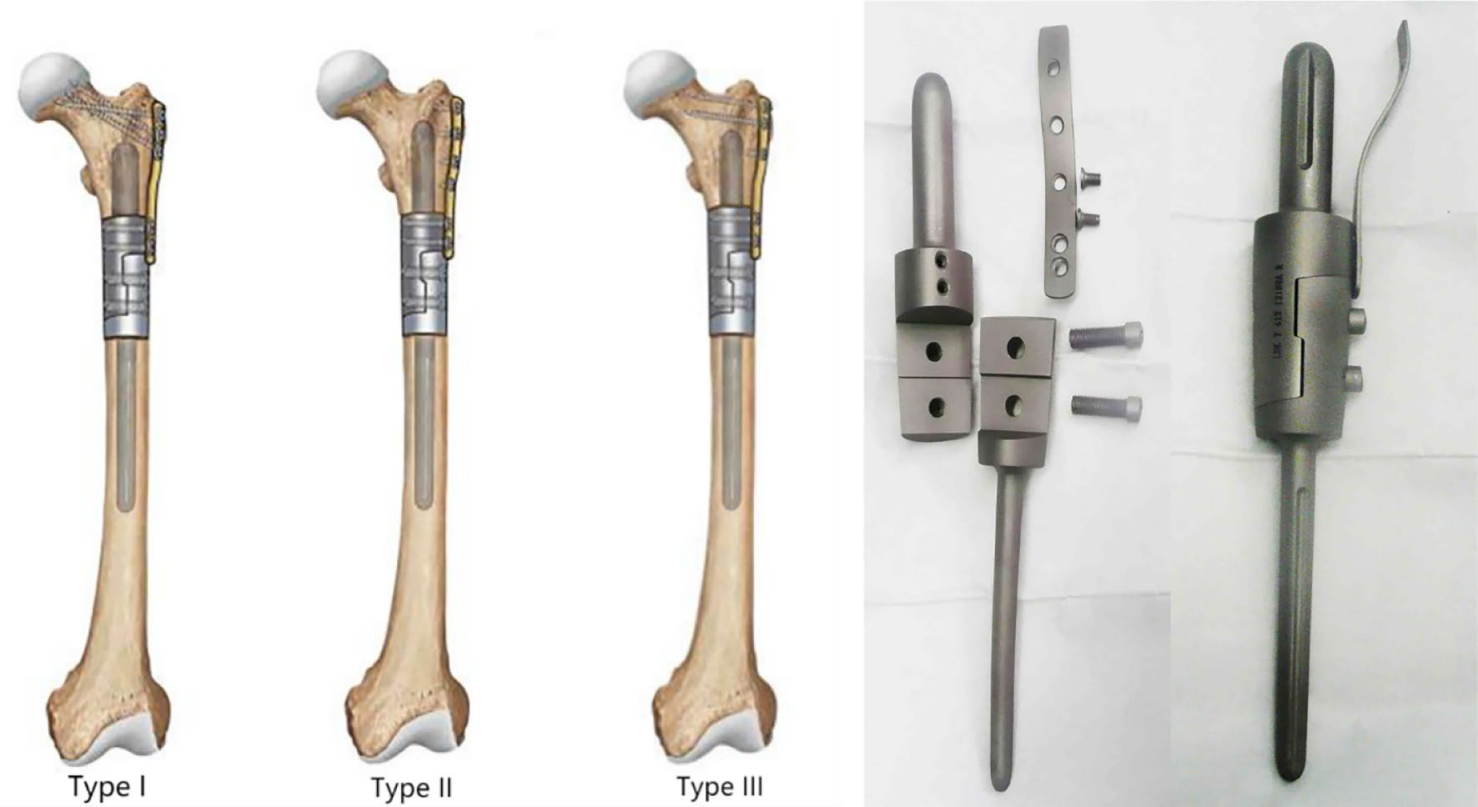
In our study, fixation classification of auxiliary lock plate includes three types: Type I (fixation through femoral neck), Type II (unilateral cortical fixation), Type III (fixation through greater trochanter).

In our study, fixation classification of auxiliary lock plate includes three types: Type I (fixation through femoral neck), Type II (unilateral cortical fixation), Type III (fixation through greater trochanter) (Fig. 6).

#### Prostheses and screws measurement

**Figure 7:**
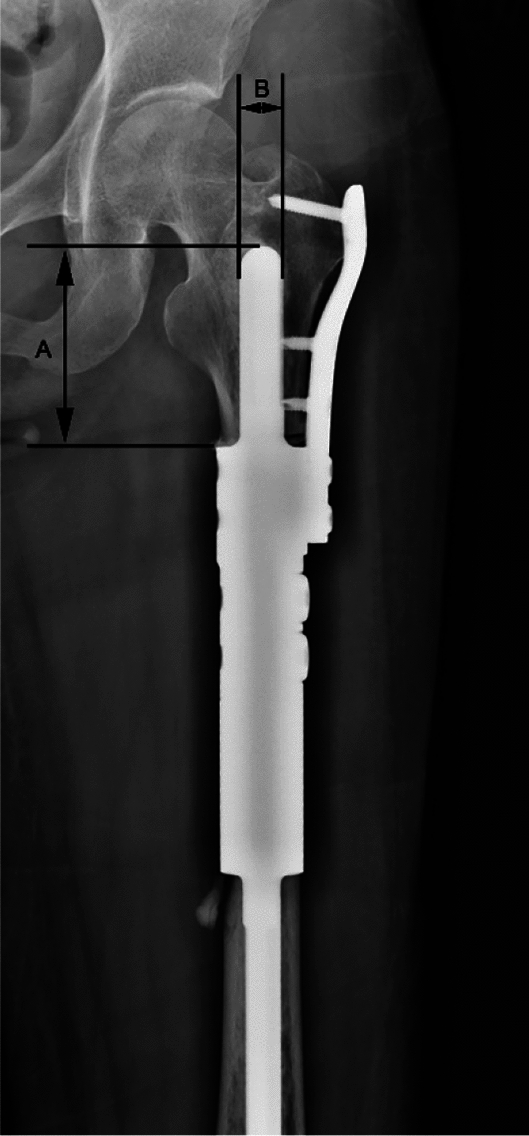
(**A**) The distance from the apex of stem to reconstruction segment is the length of proximal stem; (**B**) The diameter of the proximal stem.

Proximal stem refers to the prosthesis fixed in the medullary cavity of proximal femur. The length and diameter of the proximal stem were measured on PR (Fig. 7), and the number and location of screws for fixing auxiliary lock plate were obtained from PR. From the above results, to discover the relationship between prosthesis complications and them.

#### Resected bone and residual bone measurement

**Figure 8:**
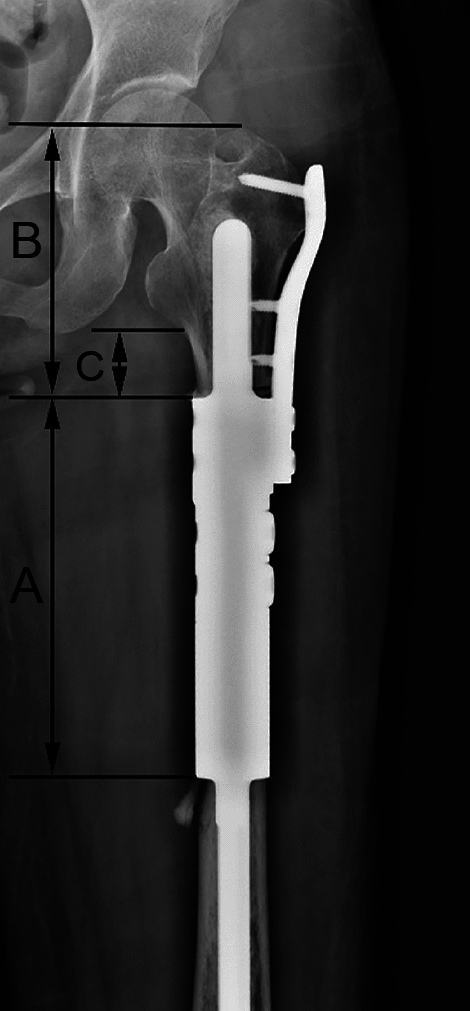
(**A**) The length of reconstruction segment is the resected bone length; (**B**) The distance from proximal end of the defect to the apex of greater trochanter is the residual bone length; (**C**) The distance from proximal end of the defect to the lower edge of lesser trochanter.

The length of residual bone refers to the remaining proximal femur length after resecting the tumour. The length of resected bone, residual bone, and the distance from proximal end of the defect to the lower edge of lesser trochanter were measured on PR to discover the relationship between prosthesis complications and them (Fig. 8).

#### Oncological outcome

Oncological outcome was used to evaluate survivorship of the patient, recurrence and metastasis of the tumor. The oncological outcome was assessed in terms of tumor entity and survival of patients, including three categories: died of the disease; no evidence of disease; and alive with evidence of disease, including alive with metastasis, alive with recurrence, alive with metastasis and recurrence.

### Functional results

#### MSTS score

The Musculoskeletal Tumour Society (MSTS) scoring system was used to evaluate postoperative functional outcomes of patients. It contained six categories including pain, activity and restriction level, emotional acceptance, use of orthopedic braces, walking ability and gait. Each of the variables was measured on a 5-point scale. The maximum score was 30 points and recorded as 100%, and higher the score or percentage, better the functional result.

#### VAS score

The visual analog scale (VAS) was a subjective index for measuring the severity of pain, a 100-mm horizontal line with the anchors was used to score that 0 mm was defined as “no pain” and 100 mm as “worst imaginable pain”. The VAS score was used to compare the changes of pain between preoperative and postoperative periods. A score of 1–3 represents that the pain is relatively mild and can be tolerated by the patient; a score of 4–6 indicates that the sleep will be affected by the pain, but can also be tolerated; a score of 7–10 indicates that the patient has gradually strong pain and can't tolerate it.

### Qualification control

To minimize the variations between observers, all measurements were taken twice separately by two experienced authors who did not know the functional results of patients and each other’s measurement results. To compare the measurements taken by two authors, the concordance correlation coefficient was used to assess interobserver agreement. The mean results of the two authors were used for the final analysis. The radiographs were measured twice by another senior author if the measurements are inconsistent.

### Statistical analysis

The continuous variables, including the VAS and MSTS score, prosthesis parameters, resected bone length and residual bone length, were presented as mean ± standard deviation. The categorical variables, including the postoperative complications and survivorship, were expressed as counts with percentages. P value less than 0.05 was considered statistically significant. SPSS 26.0 software (IBM, Armonk, NY, USA) was used for statistical analysis.

## Data Availability

All supporting data are available based on reasonable request from the corresponding author.
